# Outcomes of vaginal hysterectomy for uterovaginal prolapse: a population-based, retrospective, cross-sectional study of patient perceptions of results including sexual activity, urinary symptoms, and provided care

**DOI:** 10.1186/1472-6874-9-9

**Published:** 2009-04-20

**Authors:** Mojgan Pakbaz, Ingrid Mogren, Mats Löfgren

**Affiliations:** 1 Department of Clinical Science, Obstetrics and Gynecology, Umeå University, Umeå, Sweden

## Abstract

**Background:**

Vaginal hysterectomy is often used to correct uterovaginal prolapse, however, there is little information regarding outcomes after surgery in routine clinical practice. The objective of this study was to investigate complications, sexual activity, urinary symptoms, and satisfaction with health care after vaginal hysterectomy due to prolapse.

**Methods:**

We analyzed data from the Swedish National Register for Gynecological Surgery (SNRGS) from January 1997 to August 2005. Women participating in the SNRGS were asked to complete surveys at two and six months postoperatively. Of 941 women who underwent vaginal hysterectomy for uterovaginal prolapse, 791 responded to questionnaires at two months and 682 at six months. Complications during surgery and hospital stay were investigated. The two-month questionnaire investigated complications after discharge, and patients' satisfaction with their health care. Sexual activity and urinary symptoms were reported and compared in preoperative and six-month postoperative questionnaires.

**Results:**

Almost 60% of women reported normal activity of daily life (ADL) within one week of surgery, irrespective of their age. Severe complications occurred in 3% and were mainly intra-abdominal bleeding and vaginal vault hematomas. Six months postoperative, sexual activity had increased for 20% (p = 0.006) of women and urinary urgency was reduced for 50% (p = 0.001); however, 14% (n = 76) of women developed urinary incontinence, 76% (n = 58) of whom reported urinary stress incontinence. Patients were satisfied with the postoperative result in 93% of cases and 94% recommended the surgery.

**Conclusion:**

Vaginal hysterectomy is a patient-evaluated efficient treatment for uterovaginal prolapse with swift recovery and a low rate of complication. Sexual activity and symptoms of urinary urgency were improved. However, 14% developed incontinence, mainly urinary stress incontinence (11%). Therefore efforts to disclose latent stress incontinence should be undertaken preoperatively.

## Background

In Sweden, at least 15% of women over the age of 40 years suffer from vaginal prolapse or urinary incontinence [1-3]. The incidence of pelvic organ prolapse increases with age, and by age 80 a woman's lifetime risk of undergoing a single operation for prolapse or urinary incontinence is 11%, with a 29% risk of reoperation [4]. As the population ages, the need for medical care for uterine or vaginal prolapse will probably increase.

Vaginal hysterectomy is one of several surgical procedures for the correction of symptomatic prolapse. Reported benefits of vaginal hysterectomy compared to abdominal hysterectomy in the U.S. include shorter duration of hospital stay (WMD 1.0 day, 95% CI: 0.7 to 1.2 days), speedier return to normal activities (WMD 9.5 days, 95% CI: 6.4 to 12.6 days), and fewer unspecified infections or febrile episodes (OR 0.42, 95% CI: 0.21 to 0.83) [5].

To the best of our knowledge no published study has investigated the process of normal recovery in a routine health care setting after vaginal hysterectomy due to prolapse. The overall aim of this study was to describe the course of events after vaginal hysterectomy as a treatment for uterovaginal prolapse. The specific aims of the study were to investigate operative and postoperative complications, perceived health, sexual activity, urinary symptoms, and patient satisfaction with health care.

## Methods

### The Swedish National Register for Gynecological Surgery

In 1997, the Swedish National Register for Gynecological Surgery was established and started to collect preoperative, perioperative, and postoperative information on women undergoing hysterectomy for non-malignant pathology. The register has been used in other studies [6-8] and covers approximately 60% of the Swedish female population. In 1997, 11 of 56 departments of gynecology and obstetrics in Sweden participated in the register; by 2005, 34 different gynecological units participated. Data was collected through gynecologist forms and patient questionnaires (Table 1). The patient questionnaires were designed, constructed, and validated by the Department of Educational Measurement, Umeå University, Sweden. Three questionnaires (one preoperative, one two months postoperative, and one six months postoperative), were sent to the patients and returned to the gynecologist for evaluation. The answers were later pooled into a central database.

**Table 1:** Description of the sources of information in the Swedish National Register for Gynecological Surgery.

**Questionnaire**	**Collection time of questionnaire**	**Questionnaire data content**
Preoperative patient questionnaire (QP)	At decision for surgery	Sociodemographic data, health status, and medical assessment of patient's health data and symptoms reported by the patients

Preoperative form (Gynecologist)	At decision for surgery	History; physical and gynecological examinations

Operation form (Gynecologist)	Directly after surgery	Surgery data

Postoperative form (Gynecologist)	After discharge	Course of events during hospital stay

Two-month follow-up questionnaire answered by the patients (Q2)	Sent 6 weeks after surgery. Usually completed and registered approximately 8 weeks postoperative. 1st reminder is sent after 2 months + 3 weeks; 2nd reminder is sent after 2 months + 6 weeks.	General and medical follow-up questions, wellbeing and surgery-related complications, recovery, ratings of satisfaction and improvement; gynecologist assesses patients' answers

Six-month follow-up questionnaire answered by the patients (Q6)	Sent 6 months after surgery. 1st reminder is sent after 6 months + 3 weeks; 2nd reminder is sent after 6 months + 6 weeks.	Identical questions as in QP for symptoms (urinary symptoms, dyspareunia) and sexual activity; gynecologist assesses patients' answers

On the preoperative patient questionnaire (QP), patients responded to detailed questions designed to classify, evaluate, and grade different diseases and symptoms, such as "sensation of vaginal heaviness and/or protrusion/prolapse" and urinary incontinence. Patients' self-reported data concerning general health information were also collected on the QP. The preoperative surgeon's form included questions concerning physical and gynecological examinations of the patient.

The two-month follow-up patient questionnaire (Q2) contained general and medical follow-up questions concerning patient-reported well-being, surgery-related complications, recovery, and patient's ratings of satisfaction and improvement. Questions about short-term complications following surgery were mainly included on the Q2 questionnaire.

In the two-month follow-up questionnaire (Q2), patients were asked to report complications occurring *after *the hospital stay. Nevertheless, approximately 25% of major complications occurring *during *the hospital stay were reported in the Q2. The Q2s were evaluated and assessed by the responsible surgeons. The severity of postoperative complications was evaluated by both the patient and the gynecologist.

The Q2 questionnaires were sent to the patients by mail six weeks after surgery and were usually completed and registered approximately eight weeks postoperative. First and second reminders, when necessary, were mailed out approximately 11 and 14 weeks after surgery. (Table 1).

The six-month follow-up patient questionnaire (Q6) included some questions identical to those on the QP concerning sexual activity and urinary incontinence, as well as questions about long-term complications, such as dyspareunia, as opposed to the short-term complications asked about on Q2.

### The sample

**Figure 1:**
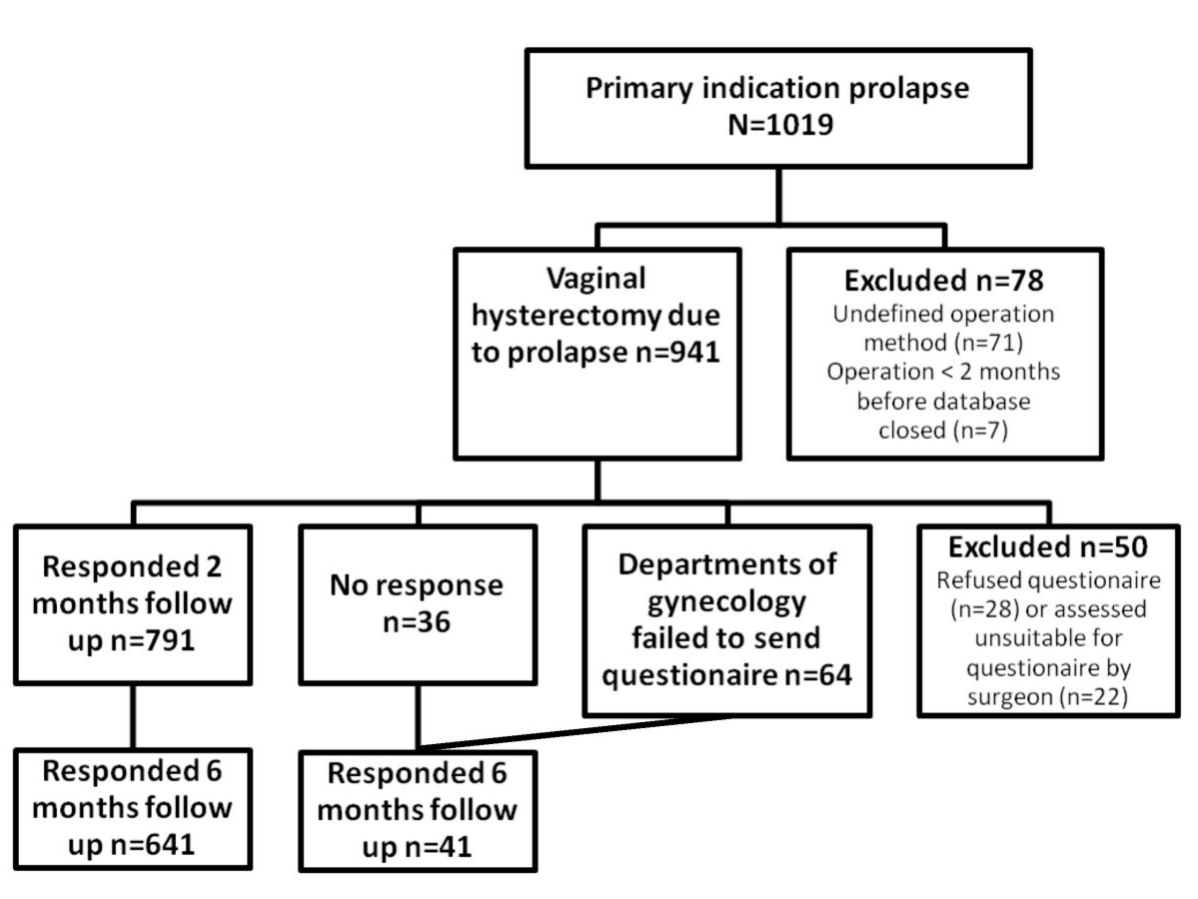
**Flow diagrams of selection of subjects eligible for the study**.

We conducted a retrospective, cross-sectional, population-based study using the SNRGS, which includes data on all registered women who underwent vaginal hysterectomy for uterovaginal prolapse from January 1997 to August 2005. All levels of health care centers, from local clinics to national university hospitals participated. The inclusion criteria was vaginal prolapse with planned correction through vaginal hysterectomy (n = 941). Exclusion criteria were surgical correction other than vaginal hysterectomy (n = 71) and surgery less than two months before closure of the database (n = 7). Women who refused the postoperative questionnaires (n = 28) and those who were assessed by their surgeons as unsuitable for questionnaires due to difficulty with Swedish language, mental disorder, or advanced age (n = 22) were excluded from the follow-up study. The sample is presented in Figure 1. Variables used in the study were collected from the SNRGS.

In total, 181 gynecologists were registered as responsible surgeons for 4664 vaginal hysterectomies for all reasons; 941 of these operations were performed due to prolapse. The range of registered number of operations per surgeon was 1–49, and the median number was 3.

The Q2 was completed and returned by 791 patients; of the 150 patients who did not complete the Q2, 64 had not received the questionnaire.

Of 941 patients who had vaginal hysterectomy due to prolapse, 827 received the Q2 questionnaire and 791 (95.5%) responded (Figure 1). Only 36 patients who received the questionnaire did not respond. Patients were routinely sent two reminders, but only four of the non-responding patients received both reminders; the other 32 received no reminder (n = 20) or only one reminder (n = 12). Non-respondents' medical records (n = 36) were retrieved after contact with the gynecological units of the responsible hospitals. We found that among patients who were not reminded at all, one subject was exposed to a severe complication (intra-abdominal hematoma) during hospital stay, which was subject to reoperation and was reported in the postoperative form by the surgeon. Two mild complications (urinary tract infection and vaginal vault hematoma with spontaneous drainage) were noted as well.

Of 941 patients who had surgery, 748 received the Q6 questionnaire and 682 responded (91%). Fifty women were excluded from receiving both Q2 and Q6 (Figure 1). Of 891 patients suitable for the Q6, 82 subjects were not yet six months postoperative at the closure of the database. There were 61 women to whom no questionnaire was sent, due to failure in hospital routines.

### Statistics

All statistical analyses were performed using SPSS 11.5. Categorical variables were analyzed by Pearson's chi-square or Fisher exact test and parametric data were analyzed by *t*-test. Holm's corrected Bonferroni method was used to correct for multiple testing. A *p-*value less than 0.05 was considered significant. The study was approved by the Ethics Committee University of Umeå, Sweden (Dnr 08-076).

## Results

Background characteristics of both respondents and non-respondents to the two-month questionnaire are summarized in Table 2. Using the Bonferroni adjustment for multiple tests we found no differences between the groups.

**Table 2:** Test of difference for specified background variables for respondents and non-respondents.

**Variable**	**Respondents**	**Non-respondents**	**P-value**
**Preoperative forms distributed (%)**	**782 (98.7)**	**125 (83.3)**	
Mean age in yrs (SD; n)	63.6 (11; 782)	62.1 (11.9; 125)	
Age group			
< 50 years n (mean age yrs)	91 (44.1)	17 (42.4)	0.952
> 50 years n (mean age yrs)	700 (66.1)	133 (65.4)	
Mean parity (SD; n)	2.47 (1.14; 741)	2.44 (1.27; 102)	0.799
Mean BMI (SD; n)	25.96 (3.6; 720)	26.3 (3.6; 102)	0.383
Mean weigh in kg (SD; n)	69.46 (10.75; 732)	70.18 (10.76; 104)	0.519
Mean height in cm (SD; n)	163.4 (6.0; 735)	163.1 (5.6; 103)	0.602
Patient's first rank of discomfort is prolapse			
n (mean)	750 (1.24)	105 (1.26)	0.771
Hypertension			
yes % (n)	32 (250)	29.6 (37)	0.597
don't know % (n)	68 (532)	70.4 (88)	
Current disease other than gynecologic			
Yes % (n)	65.9 (396)	69.3 (61)	0.525
no % (n)	34.1 (205)	30.7 (27)	
Diabetes mellitus			
yes % (n)	5.8 (35)	14.8 (13)	0.002
no % (n)	94.2 (566)	85.2 (75)	
Asthma			
Yes % (n)	4.2 (25)	10.2 (9)	0.014
no % (n)	95.8 (576)	89.8 (79)	
Current use of estrogen			
yes % (n)	34.3 (192)	28.4 (23)	0.294
no % (n)	65.7 (368)	71.6 (58)	
Smoking			
yes % (n)	12.5 (74)	18.4 (16)	0.127
no % (n)	87.5 (520)	81.6 (71)	
Registration forms distributed* (%)	**791 (100)**	**150 (100)**	0.947
Mean uterine size estimated as corresponding gestational age (SD; n)	3.27 (1.66; 775)	3.28 (1.63; 143)	
ASA I† % (n)	88.9 (518)	80 (68)	0.020
ASA II† or more % (n)	11.1 (65)	20 (17)	
**Number of operation forms available (%)**	**791 (100)**	**150 (100)**	
Mean duration time in minutes (SD; n)	76.61 (29.7; 778)	77.84 (30.1; 143)	0.652
Mean blood loss in ml (SD; n)	127.38 (131.3; 757)	128.86 (129.7; 138)	0.903

(Student's *t*- test for parametric data and Pearson's chi-square test for categorical data).

SD, Standard deviation; n, number of patients in each group.

* Registration forms were completed by the surgeon when decision for surgery was made.

† American Society of Anesthesiologists Physical Status Classification System.

### Operative and short-time post-operative results

Vaginal hysterectomy was the single surgical procedure performed in 746 (79.3%) patients. Additional operative procedures, such as a repair of a cystocele or a rectocele, were identified in 154 cases (16.4%), of which eight cases were surgery with Tension-free Vaginal Tape (TVT) because of urinary incontinence. Information about concomitant surgery was missing in 41 subjects.

Mean estimated operation time was 76 minutes (95% CI: 74–78) and the mean blood loss volume was 128 ml (95% CI: 119–136).

Conversion to laparotomy was performed in two cases, one due to complication because of bleeding and the other because of adhesions, narrow vagina, and immobile uterus. Reoperation during hospital stay occurred in about 1% of patients (Table 3).

**Table 3:** Reoperations during hospital stay and complications resulting in readmission.

**Reoperations during hospital stay**	**n**	**Treatment**
Retroperitoneal or intra-abdominal bleeding	5	Reoperation
Bleeding from suture	2	Resuture
Vaginal vault abscess	1	Drainage and antibiotic
Urinary retention, due to combined TVT	1	Cutting TVT band
		
**Complications resulting in readmission**		
Thrombosis	1	Low molecular heparin
Ileuses	1	Laparotomy, resection of small bowel
Vaginal vault abscess	8	Drainage and antibiotic or antibiotic only
Urinary retention, UTI	2	Catheter and revisit, antibiotics
Arthritis in knee joint, Allergic reaction	2	Consultant internal medicine

The discharging gynecologist evaluated the operation and postoperative hospital stay as without complications in 91% of cases, with minor complications in 7.5% of cases, and with severe complication in 1%; missing information constituted 0.5% (n = 22). Among 22 patients without discharge data, 17 patients reported a recovery without complications in the two-month follow-up questionnaire. For the remaining five patients, medical files were retrieved and no complications were reported.

The prevalence of infections associated with the operative procedures and occurring during hospital stay was estimated at 2% for vaginal infections, 1.3% for urinary tract infections (UTI), and 0.1% for vaginal infections and UTI; 96.6% of women did not develop an infection.

Most patients (95%) received preoperative antibiotics and infection was reported in 1.3% (n = 11) of them. Infection was not observed among the 47 women who did not receive preoperative antibiotic prophylactic. This difference was not statistically significant (p = 0.403).

All major complications during the hospital stay that were reported by the patients on the Q2 were also reported by the surgeon on the postoperative form.

### Postoperative results reported by respondents two months after surgery

Of 791 patients who completed the Q2, 17 patients reported ensuing complications that required their re-admission to the hospital. Three reported complications that occurred during the hospital stay and 14 experienced complications after discharge requiring readmission, of which most were infections (Table 3).

There were 23 (2.9%) patients who required reoperation during the hospital stay and readmission. There were no statistically significant differences between these patients and the rest of the study population regarding medication, preoperative functional state according to the American Society of Anesthesiologists Physical Status Classification System (ASA), unexpected operation difficulties, such as adhesions or presence of endometriosis, or perioperative complications (Table 4). Women who had complications were younger (p = 0.001) and had fewer concurrent diseases (p = 0.007) than subjects with no complications.

**Table 4:** Specified variables compared between patients with complications and patients with no complications.

**Variable**	**Uncomplicated**	**Complicated**	**P-value**
Mean age in years	64	56	0.001
Mean BMI	26	24.5	0.034
Current disease beside gynecologic %	67	37	0.007
Current medication, %	66	50	0.132
ASA I %	85	85	0.982
No surgical difficulties, %	90	95	0.373
No perioperative complications, %	97	100	0.419
Mean operation time, min	77	65	0.003
Mean blood loss, ml	126	162	0.8

Student's *t*-test for parametric data and Pearson's chi-square test for categorical data.

Of 23 complications that resulted in reoperation or readmission, 19 (82.6%) complications occurred in subjects who had solely vaginal hysterectomy and 4 (17.4%) in subjects who had concomitant surgery. The difference was not statistically significant (p = 0.93).

Patient-reported infections after discharge that required antibiotics were vaginal infections in 3.9% (n = 31), lower UTI in 5.6% (n = 44), and UTI and vaginal infection in 1.4% (n = 11). With only one exception, all cases with infections received preoperative prophylactic antibiotics. No signs of infection were reported by 89.1% of the respondents.

Table 5 shows other complications reported by the patients. Patients sought treatment for complications from gynecological departments (8.8%) and general practitioners (4.7%).

**Table 5:** Other complications reported by the patients.

**COMPLICATIONS**	**REPORTING %**
Conservatively treated intra-abdominal abscess*	0.8
Conservatively treated vaginal vault abscess*	4
Urinary retention	0.3
Fever > 38°C for two or more days*	4.3
Deep vein thrombosis	0.1
Fatigue	**7**.2

* Not receiving antibiotics.

More than 90% of patients rated the length of the hospital stay as adequate; the median hospital stay was 3 days, with 44% of patients discharged within 2 days postoperatively. Patients' estimated time of return to normal activity of daily life (ADL) showed no difference between age groups (p = 0.093; Table 6).

**Table 6:** Patients' self-estimated time to recurrence to normal daily activity.

	**Age (years)**				
	< 40	40–50	50–60	>60	Total
	(n = 11)	(n = 71)	(n = 162)	(n = 350)	(n = 594)
**Days**	%	%	%	%	
0–2	0	12.7	13.6	12	12.3
3–7	100	62	59.3	53.1	56.7
8–10	0	8.5	9.9	16	13.1
>11	0	16.9	17.3	18.9	17.8

In the two-month follow-up questionnaire, 32% reported that a next visit was planned at discharge and was carried out in 91% of those cases.

Of all women who answered the Q2, 76% reported that they did not need any further contact with the gynecological department, 8.5% wished contact with the surgeon, 11.9% asked for a return visit, and 3.5% did not respond to the specific question.

From patients' postoperative questionnaires, surgeons assessed no further intervention for 82%, a return visit for 9.7%, and telephone contact for 6.0%; information was missing for 2.4%.

### Evaluations of complications by surgeons compared with patients

Generally, patients and surgeons agreed about whether there had been an adverse postoperative event (73%). When opinions differed, patients tended to evaluate their postoperative recovery as more complicated than their surgeons did (15.7%), although the opposite evaluation occurred in 1.7% (Table 7). Table 8 shows the discrepancy in evaluation of severe complications between surgeons and patients.

**Table 7:** Surgeons' and patients' evaluation of postoperative course of events.

		**Patients' evaluation of postoperative events**				
		
		No complication	Minor complication	Severe complication	Missing information	Total answers
		n (%)	n (%)	n (%)	n (%)	n (%)
**Surgeons' evaluation of postoperative events**	No complication	484 (61)	115 (14.5)	1 (0.13)	26 (3.3)	626 (79)
	Minor complication	9 (1.14)	83 (10.5)	8 (1)	0	100 (12.6)
	Severe complication	2 (0.25)	3 (0.38)	6 (0.76)	0	11 (1.4)
	Missing information	30 (3.8)	14 (1.8)	6 (0.76)	4 (0.5)	54 (6.8)
	Total answers	525 (66.3)	215 (27)	21 (2.65)	30 (3.8)	791 (100)

**Table 8:** Discrepancy in the evaluations of severe complications between surgeons and patients.

**Surgeon**	**Patient**	**Complications reported at two-month follow up**
Severe	None	Perioperative paresis of the peroneal nerve*
Severe	None	Intra-abdominal bleeding*
Severe	Mild	Two cases of intra-abdominal bleeding* and drainage of vaginal abscess
Severe	Mild	Thrombosis treated with low molecular heparin
None	Severe	Upper UTI 6 weeks postoperative
Mild	Severe	Urinary incontinence that might have increased after operation
Mild	Severe	Vaginal vault abscess 3 weeks postoperative, treated with antibiotics and drainage
Mild	Severe	Dyspareunia because of narrow vagina
Mild	Severe	Upper UTI two months postoperative
Mild	Severe	Vaginal vault infections, 4 cases, 1–3 weeks postoperative, treated with antibiotics

*Complication occurred during hospital stay.

Both surgeons and patients unanimously considered complications as mild in 10.5% (n = 83) of cases, and 51% of reported complications (n = 42) were treatable infections such as UTI and bacterial vaginosis. The remaining complications were relatively minor conditions, such as fatigue, pain, urethritis, and constipation.

Of the 115 patients who reported minor complications while their surgeons reported no complications, 67% (n = 77) did not seek further medical care.

Ninety-seven per cent of the women reported that their condition had been improved. The operation was recommended by 94% of the women.

### Results reported by respondents six months after surgery compared with preoperative reports

Only women who responded to both the preoperative questionnaire (QP) and the Q6 were investigated. Patients were asked to answer in both questionnaires the specific question, "Did you have coitus in the last three months?" Of 244 women who had coitus within three months prior to surgery, 6.6% (n = 16) of them had not resumed coitus within six months postoperative. Among the 376 women who had not had coitus three months prior to surgery, 17% (n = 64) had resumed coitus six months postoperative. The number of women reporting intercourse had increased by 20% from 244 to 292 cases (p = 0.006). A total of 9% (n = 62) did not respond to the specific question in the preoperative or the six-month postoperative questionnaire.

Dyspareunia was evaluated by the patients with a visual analogue scale (VAS) in the preoperative and six-month postoperative questionnaires. The scale measured from 0 to 50 mm where 0 equalled complete absence of symptoms and 50 represented unbearable symptoms. The mean value for dyspareunia according to the VAS was reported as 0.90 mm preoperatively and 0.84 mm postoperatively. No statistical significant difference was found (p = 0.072, 95% CI: 0.022–0.522).

Of 682 women who answered the Q6, 679 (99.5%) answered questions regarding urinary symptoms, such as urgency, urge incontinence, or urinary stress incontinence in both the QP and the Q6. In the preoperative questionnaire 38.1% of the respondents (n = 259) reported urinary problems; six months postoperatively this response decreased to 30% (n = 204) (p = 0.002). Fourteen per cent (76 of 545; Table 9) of women who were continent prior to surgery became incontinent postoperatively, mainly with urinary stress incontinence (n = 58). Conversely, of 134 women who reported urinary incontinence in the QP, 51.5% (n = 69) became continent postoperatively; 134 women were incontinent preoperative (19.7%) and 141 postoperative (20.8%; p = 0.685). Urgency was reduced by 50.4% (p < 0.001; Table 9).

**Table 9:** Urinary symptoms reported by patients, preoperative and six months postoperative.

	**6 months postoperative**				
	
**Preoperative**	No reported symptoms	Urgency	Urge incontinence	Stress incontinence	Total
No reported symptoms n (%)	336 (49.5)*	31 (4.6)^†^	11 (1.6)^‡^	42 (6.2)^§^	420 (61.8)*
Urgency n (%)	78 (11.5)	24 (3.5)	7 (1)^‡^	16 (2.4)^§^	125 (18.4)^†^
Urge incontinence n (%)	40 (5.9)^‡^	6 (0.88)	15 (2.2)	23 (3.4)	84 (12.4)^‡^
Stress incontinence n (%)	21 (3)^§^	2 (0.3)	4 (0.4)	23 (3.4)	50 (7.4)^§^
Total, n (%)	475 (70)	63 (9.3)^†^	37 (5.4)^‡^	104 (15.3)^§^	679 (100)

*Of respondents without preoperative urinary symptoms, one fifth reported urinary symptoms postoperatively.

^†^Urgency is reduced in about half of those reporting it preoperatively, but half of the respondents with postoperative urgency had no preoperative symptoms.

^‡^Urge incontinence is reduced by about 60%, however almost half of women with postoperative urge incontinence were continent preoperatively.

^§^Number of women with postoperative stress incontinence doubled compared to number preoperative.

Sensation of vaginal heaviness or pressure was rated by the patients with a visual analogue scale (VAS) in the QP and the Q6. The scale measured from 0 to 50 mm where 0 equalled complete absence of symptoms and 50 represented unbearable symptoms. Of women who rated their symptoms on a VAS scale as above 80% of the scales length (n = 262) preoperatively, 73.3% expressed no symptoms of heaviness or pressure on the six month follow-up (p < 0.001, 95% CI: 2.32–2.63) and 81.3% (n = 620) had no symptoms regardless of previous grade of heaviness. There were 62 women who did not respond the specific question in the QP or the Q6.

Respondents reported that they were satisfied with the result of the treatment in 93% (n = 629) of cases.

## Discussion

The current study was a population-based study and hospitals at every level from local, county-run to national, university-affiliated, participated. It covers 65% of all Departments of Gynecology in Sweden and approximately 60% of the Swedish female population. The main finding of our study is that vaginal hysterectomy performed for vaginal prolapse in routine clinical health care setting results in short hospital stay, swift recovery, and a low rate of complications. Furthermore, there is a high grade of patient satisfaction and the results are comparable to those of other studies of vaginal hysterectomy regardless of indication [5,9].

Retrospective data may imply certain limitations. Weaknesses of the study include lack of information in the register regarding the stage of the prolapse and the fact that decisions to perform vaginal hysterectomy for prolapse were made by individual surgeons. The register was first developed to record hysterectomies for non-malignant pathologies including prolapse. Therefore the frequency of additional prolapse surgery (cystocele, rectocele) in the registry is not representative of its frequency in the population.

The aim of the study was not to assess the anatomical success of the surgery, but rather to investigate patient-reported perceptions postoperatively. The use of postoperative patient questionnaires has previously been validated and has been found to be highly accepted by patients; furthermore, questionnaires provide more complete and thorough collection of postoperative information than follow-up visits [10].

The response rate in the patients' postoperative questionnaires (at two and six months) was very high. Non-response in the postoperative questionnaire was mainly related to flaws in participating hospitals' procedures for sending the reminders. If the questionnaires (with two reminders) were sent by surgeons responsible for the patients, the response rate exceeded 95%. We did not find any complications during hospital stay that were reported by the patients but not registered by the surgeons. Thus, the data from the SNRGS used in the current study was without any greater bias or flaw.

A low rate of severe complications (3%) was found; these were mainly intra-abdominal bleeding and vaginal vault hematomas or infections. Surprisingly, there were no recorded bladder injuries or perforations during surgery.

In a meta-analysis review article, the rate of complications requiring reoperation after apical vaginal prolapse surgical repair was highest for vaginal mesh kit compared to traditional vaginal surgeries and sacral colpopexy [11]. In Sweden apical suspension to the sacrouterine ligament is an integral part of vaginal hysterectomy when performed for prolapse, but individual data on apical suspension was not available for the present study.

In the present study because the frequency of complications did not differ between women who had vaginal hysterectomy only and those who had another concomitant procedure (p = 0.93), concomitant procedures did not seem to increase the risk for complications. However, since the registry database does not record the type of apical suspension used, we can not conclusively exclude additional procedures as possible additional risk factors for complications.

UTI and vaginal infections dominated (50%) mild complications. The main difference between patients with and without complications was age and the majority of the complications were attributed to infections. Likewise, younger patients have an increased risk for postoperative infection [12].

The patients reported more complications, and of greater severity, in the postoperative course than the surgeons did. Disagreements mostly arose when patients reported complications and surgeons did not or were unaware [13]. We believe patients evaluated the severity of complications based on their experience of discomfort, while surgeons evaluated complications based on the level of medical risk. Patients and surgeons had different frames of reference for risk assessment.

The finding in patients' self-assessment of recovery to ordinary activity of daily life was not highly affected by age or general health, presumably because the patients compared their postoperative activity with their normal preoperative functioning.

The number of women who were sexually inactive prior to surgery and resumed sexual activity after surgery was four times higher than women who were sexually active prior to surgery and had not resumed sexual activity by six months postoperatively. Overall we found that vaginal hysterectomy for prolapse had a positive effect on sexual activity.

Prolapse is often accompanied by urinary symptoms [14,15]. An earlier study has shown that urinary frequency, urgency, and urge incontinence disappeared in 60%, 70%, and 82% of women respectively one year after the repair of anterior vaginal wall prolapse [16]. In this study, while symptoms of urgency and urge incontinence were reduced by half, the proportion of women with urinary stress incontinence (USI) doubled postoperatively. Not uncommonly, pelvic organ prolapse may lead to latent USI [15]. In the current study almost 11% (58/545) of respondents who were continent prior to surgery reported urinary stress incontinence six months postoperatively. Performing urodynamic investigations in patients undergoing prolapse surgery [17] and ring pessary reduction of severe prolapse during urodynamics [15] has been shown valuable for diagnoses of USI or occult USI. Efforts to disclose latent stress urinary incontinence should be undertaken preoperatively.

Further studies are needed, particularly relating to long-term results after prolapse surgery.

## Conclusion

Vaginal hysterectomy is a patient-evaluated efficient treatment for uterovaginal prolapse with a swift recovery and a low rate of complications. Sexual activity and symptoms of urinary urgency were improved. However, 11% of the respondents developed urinary stress incontinence. Therefore efforts to disclose latent stress incontinence should be undertaken preoperatively.

## Abbreviations

ADL: Activity of daily life; ASA: American Society of Anesthesiologists Physical Status Classification System; BMI: body mass index; CI: confidence interval; OR: odds ratio; QP: preoperative questionnaire; Q2: Two-month follow-up questionnaire; Q6: Six-month follow-up questionnaire; SNRGS: Swedish National Register for Gynecological Surgery; TVT: Tension-free Vaginal Tape; USI: urinary stress incontinence; UTI: urinary tract infection; WMD: weighted mean difference.

## Competing interests

The authors declare that they have no competing interests.

## Authors' contributions

MP was involved in data collection, data analysis, and manuscript writing. ML was involved in study design, data collection, and data analysis. IM participated in the design of the study and drafting of the manuscript. All authors read and approved the final manuscript.

## Pre-publication history

The pre-publication history for this paper can be accessed here:


